# Subunit composition of respiratory chain complex 1 and its responses to oxygen in mitochondria from human donor livers

**DOI:** 10.1186/s13104-017-2863-7

**Published:** 2017-11-02

**Authors:** S. E. Khorsandi, J. W. Taanman, N. Heaton

**Affiliations:** 1 Institute of Liver Studies, King’s College Hospital, Kings College London, London, SE5 9RS UK; 2 Department of Clinical Neurosciences, Institute of Neurology, University College London, London, UK

**Keywords:** Mitochondria, Liver transplantation, Complex 1, Oxygen, Donor after cardiac death

## Abstract

**Objective:**

Donor liver function in transplantation is defined by mitochondrial function and the ability of mitochondria to recover from the sequence of warm and/or cold ischemia. Mitochondrial resilience maybe related to assembly and- subunit composition of Complex 1. The aim of this study was to determine if Complex 1 subunit composition was different in donor livers of varying quality and whether oxygen exposure had any effect.

**Results:**

Five human livers not suitable for transplant were split. One half placed in cold static storage and the other half exposed to 40% oxygen for 2 h. Protein was extracted for western blot. Membranes were probed with antibodies against β-actin and the following subunits of Complex 1: MTND1, NDUFA10, NDUFB6 and NDUFV2. No difference in steady state Complex 1 subunit composition was demonstrated between donor livers of varying quality, in terms of steatosis or mode of donation. Neither did exposure to oxygen influence Complex 1 subunit composition. This small observational study on subunit levels suggest that Complex 1 is fully assembled as no degradation of subunits associated with the different parts of the enzyme was seen.

## Introduction

Liver transplantation is limited by the lack of organs and combined with lengthening waiting lists results in a waiting list mortality of up to 20%. To address this disparity the marginal liver in terms of donor age, liver steatosis or donation after cardiac death (DCD) is increasingly used. But there is a risk to the recipient in terms of graft dysfunction, graft loss or death.

Previous work has demonstrated that mitochondrial recovery and ability to generate adenosine triphosphate (ATP) effectively is a determinant of survival after liver transplantation. Mitochondria are central to cellular health through the production of ATP by oxidative phosphorylation (OXPHOS) and reactive oxygen species (ROS) that both drive and define cell function [1]. Consequently, many of the strategies used to optimize the donor liver are aiming to limit mitochondrial injury, such as solutions for cold static storage and minimizing ischemic times (warm and cold).

The largest complex of the respiratory chain is Complex 1 (> 900 kDa). It is an L-shaped multimeric protein complex, composed of 45 subunits and is the electron entry point of the respiratory chain. Disruption in the organization of Complex 1 can lead to an energy switch from OXPHOS to glycolysis [2]. The factors that drive the formation of mitochondrial respirasomes, the functional units of respiration, composed of complex 1, 3 and 4 remain unknown, but Complex 1 assembly and activation is regarded as a critical event. Potentially, donor liver function could be defined by mitochondrial resilience, as determined by assembly and subunit composition of Complex 1.

The aim of this work was to determine if Complex 1 subunit composition in discarded human donor livers of varying quality was different, and whether composition could be modulated by oxygen exposure.

## Main text

### Methods

#### Donor human livers and the split liver model

Donor livers (n = 5) utilized in this study had been declined for both solid organ and cell transplantation. Table 1 summarizes the clinical characteristics of the donor livers used and reason for discard. The two donor liver types that were used in this study were: (1) donor after brainstem death (DBD) and (2) donor after cardiac death (DCD). In both donor types, dual aortic and portal perfusion was performed using University of Wisconsin Solution (UW) at 4 °C. In the DBD liver, organ perfusion with blood was maintained throughout the preparatory donor dissection up to cannulation. Whereas in the DCD liver, aortic and portal vein cannulation was performed after circulatory arrest. The warm ischemic time (WIT), which is unique to the DCD liver, was defined from which agonal observation occurred first, either a systolic of 50 mmHg or oxygen saturation of 70%, to aortic cannulation. The cold ischemic time (CIT) was defined as the time from the start of cold aortic perfusion to the start of oxygen exposure i.e. anterograde persufflation (A-PSF).

**Table 1:** Summary of the clinical characteristics of the donor liver studied

	Liver 1	Liver 2	Liver 3	Liver 4	Liver 5
Donor age (years)	47	60	49	55	59
Graft type	DBD	DCD	DCD	DCD	DBD
Steatosis	50%	0%	30%	20%	0%
WIT (min)	NA	24	15	20	NA
CIT (h)	8	12	12	10	10
Reason for clinical discard	Steatohepatitis with fibrosis	Perfusion concern	Steatosis	Lymphoproliferative disorder	Cholangiocarcinoma concern

Graft types studied included donor after brain stem death (DBD) and donor after cardiac death (DCD). Liver steatosis is expressed as a percentage (%) based on histological assessment of the liver using Hematoxylin and Eosin stain. The warm ischemic time (WIT) is only applicable to the DCD liver and is from the time of oxygen saturation of 70% or systolic blood pressure of 50 mmHg in the donor to the time of aortic cannulation in the donor. The cold ischemic time (CIT) for this study is from the time of aortic cannulation in the donor to the time when anterograde persufflation (O_2_ exposure) starts. *NA* Not applicable

To assess the response of the donor liver to A-PSF an experimental split model was developed. This involved performing a surgical left lateral segment (LLS) split. Producing two sections of liver for study from one donor liver with an inflow portal vein (PV) and outflow hepatic vein. A-PSF was via the PV with gaseous venting through the hepatic veins. The experimental (O_2_ exposed/A-PSF) section of donor liver was kept on ice in UW throughout, while the non-PSF or control section of donor liver was rebagged for standard cold static storage in UW on ice.

#### Electrochemical oxygen concentrator and anterograde persufflation

Oxygen enriched atmospheric air was supplied with a portable electrochemical oxygen concentrator (Giner Inc, Newton, MA, USA). For this study atmospheric air was enriched to 40% oxygen. The PV was cannulated and A-PSF was performed with 40% O_2_ via the PV at a manifold pressure of 15–20 mmHg to achieve flows of 15–20 ml/min. Persufflation was performed for 2 h. Trucut biopsies were then taken from both sides of the liver (A-PSF/experimental O_2_ exposed and non-PSF/control) for formalin fixation and snap freezing in liquid nitrogen for storage at − 80 °C.

#### Protein extraction and analysis

Frozen liver biopsies were homogenized on ice using a micropestle in a 1.5 ml microcentrifuge tube. Homogenization buffer composition is as follows: 10 mM Hepes NaOH pH 7.4, 1 mM sodium EDTA, 250 mM sucrose, 1 μg/ml pepstatin A, 1 μg/ml leupeptin and 1 mmol phenylmethanesulfonyl fluoride. After homogenization the samples were stored at − 80 °C for later use. Homogenates were further extracted in homogenization buffer containing 1.5% lauryl *n*-dodecyl β-d-maltoside (Anatrace, USA) on ice. All reagents were from Sigma-Aldrich unless otherwise stipulated. After extraction, samples were centrifuged at 13,000*g* for 10 min at 4 °C. The cytoplasmic fraction (supernatant) was transferred into pre-cooled new tubes. For quantitation of total protein the Pierce™ BCA Protein Assay Kit (Thermo Scientific, USA) was used.

For protein electrophoresis and blotting the mini-PROTEAN^®^ Tetra Vertical Electrophoresis Cell, 4-15% mini-PROTEAN^®^ TGX Stain-Free™ Precast Gels and Trans-Blot^®^ Turbo™ Transfer System (Bio-Rad Lab Ltd, UK) were used. Reference ladder was Precision Plus Protein™ Dual Color Standards (Bio-Rad Ltd, UK). 20 μg of protein was loaded per lane. The polyvinylidene difluoride (PVDF) membrane was dried overnight before blocking and probing. In brief, membranes were blocked in 5% skimmed milk/phosphate buffered saline, 0.3% Tween (PBS-T) for 1 h followed by rinsing in PBS-T. The membrane was then probed with the primary antibody in PBS-T for 2 h at room temperature unless otherwise stated.

Primary antibodies used and dilutions are as follows: β-actin, 42 kDa, 1:120,000 (abcam^®^ ab6276); NDUFB6 (distal part of membrane arm), 17 kDa, 1:1500 overnight incubation at 4 °C (abcam^®^ ab110244); NDUFV2 (distal part of matrix arm), 24 kDa, 1:1000 overnight incubation at 4 °C (proteintech™ 15301-1-AP); NDUFA10 (middle part of membrane arm), 42 kDa (abcam^®^ ab174829); and MTND1 (proximal part of membrane arm), 36 kDa (proteintech™ 19703-1-AP).

Appropriate secondary antibodies (Dako Agilent Pathology Solutions polyclonal goat anti-mouse—P026002-2 or anti-rabbit IgG-HRP—P044801-2) were used, diluted 1:3000 in PBS-T and incubated with the membrane for 1 h. Clarity™ Western ECL Substrate (Bio-Rad Lab Ltd, UK) was used to develop the image. Image capture, background subtraction and quantification of band signal intensity was performed using Image Lab™ Software v5.1 (Bio-Rad Lab Ltd, UK). The protein of interest was normalized to β-actin. Human nomenclature for Complex 1 subunits has been used.

#### Statistics

Where appropriate, data is expressed as a mean and standard deviation. For comparison of groups the unpaired two tailed t test was used, significance value was set as p < 0.05. Statistical analysis was performed with GraphPad Prism v7.0b (GraphPad Software, Inc. USA).

### Results

#### Donor liver demographics

Five human donor livers declined for transplantation were split, considering graft type, 2 were DBD and 3 DCD, producing 10 sections of liver for study. The mean donor age was 54 ± 5.8 years, cold ischemic time 10.4 ± 1.7 h and warm ischemic time, where applicable, was 19.7 ± 4.5 min. On Hematoxylin and Eosin histology, steatosis was not demonstrated in 2 livers and in the remaining 3, steatosis ranged from 20 to 50%. The main documented reason for donor liver decline for transplantation was pathology in the donor, identified histologically as steatosis in 2 and cancer risk in another 2. In the remaining declined liver, there were concerns about preservation perfusion (Table 1). There were no technical issues on splitting the liver or on using A-PSF. In all cases, persufflation via the PV was successful, with minimal air leak from the cut surface and effective venting of O_2_ via the hepatic vein.

#### Complex 1 subunit protein analysis

**Fig. 1:**
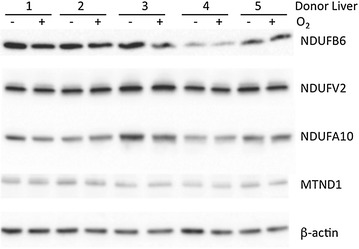
Western blot for the subunits of Complex 1. Subunits analyzed were NDUFB6 (distal part of membrane arm), NDUFV2 (distal part of matrix arm), NDUFA10 (middle part of membrane arm), MTND1 (proximal part of membrane arm) and the housekeeping β-actin protein

**Fig. 2:**
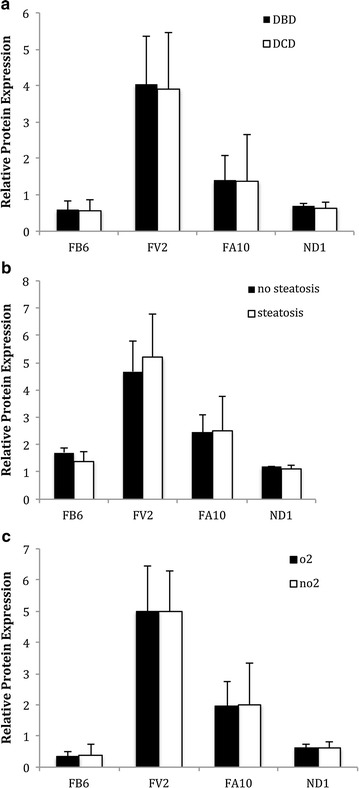
**a** Graphical summary of changes in Complex 1 subunits of the liver in donor after brainstem death v donor after cardiac death. Changes in levels of Complex 1 subunits are expressed as a ratio to β-actin comparing expression level of given subunit in donor after brainstem death (DBD, n = 4) compared to donor after cardiac death (DCD, n = 6) liver section. Complex 1 subunits compared were NDUFB6 (distal part of membrane arm), NDUFV2 (distal part of matrix arm), NDUFA10 (middle part of membrane arm) and MTND1 (proximal part of membrane arm). Y-axes represent given Complex 1 subunit signal relative to β-actin signal. Error bars indicate standard deviation. No significant difference was demonstrated. **b** Graphical summary of changes in Complex 1 subunits steatotic v non steatotic liver. Changes in levels of Complex 1 subunits are expressed as a ratio to β-actin comparing expression level of given subunit in the non steatotic (n = 4) compared to steatotic (n = 6) donor liver section. Complex 1 subunits compared were NDUFB6 (distal part of membrane arm), NDUFV2 (distal part of matrix arm), NDUFA10 (middle part of membrane arm) and MTND1 (proximal part of membrane arm). Y-axes represent given Complex 1 subunit signal relative to β-actin signal. Error bars indicate standard deviation. No significant difference was demonstrated. **c** Graphical summary of changes in Complex 1 subunits O_2_ v non O_2_ exposed section of liver. Changes in levels of Complex 1 subunits are expressed as a ratio to β-actin comparing expression level of given subunit in the section of liver exposed to oxygen (O_2_) via anterograde persufflation (n = 5) compared to the control section of liver subjected to cold static storage (n = 5). Complex 1 subunits compared were NDUFB6 (distal part of membrane arm), NDUFV2 (distal part of matrix arm), NDUFA10 (middle part of membrane arm) and MTND1 (proximal part of membrane arm). Y-axes represent given Complex 1 subunit signal relative to β-actin signal. Error bars indicate standard deviation. No significant difference was demonstrated

Figure 1 shows the western blot images for β-actin (housekeeping/reference protein) and the following subunits of Complex 1: MTND1 (proximal part of membrane arm), NDUFA10 (middle part of membrane arm), NDUFB6 (distal part of membrane arm) and NDUFV2 (distal part of matrix arm). On comparison of studied subunits according to liver section graft type, DBD (n = 4) versus DCD (n = 6) there was no difference in protein expression (NDUFB6, p = 0.53; NDUFV2, p = 0.72; NDUFA10, p = 0.75; MTND1, p = 0.48; Fig. 2a). Similarly, no difference in subunit composition was seen when comparing the steatotic (n = 6) to non steatotic (n = 4) donor liver section (NDUFB6, p = 0.81; NDUFV2, p = 0.59; NDUFA10, p = 0.89; MTND1 p = 0.28; Fig. 2b). When the effect of oxygen produced by A-PSF was analyzed, again no difference in subunit composition in Complex 1 was demonstrated on comparison of O_2_ exposed (n = 5) to non O_2_ (n = 5) exposed liver sections (NDUFB6, p = 0.36; NDUFV2, p = 0.37; NDUFA10, p = 0.93; MTND1, p = 0.52; Fig. 2c).

### Discussion

In this small observational study, no changes in Complex 1 subunit levels was demonstrated in the donor liver on exposure to oxygen when used as a resuscitative strategy after cold static storage. Suggesting that Complex 1 is fully assembled, as no degradation of subunits associated with the two arms was seen. Additionally, neither steatosis or graft type (DBD v DCD) had any bearing on Complex 1 subunit composition.

Complex 1 is a L-shaped structure composed of 45 subunits. One limb is a hydrophobic membrane arm sitting in the mitochondrial inner membrane and the other arm is hydrophilic, projecting into the mitochondrial matrix [2]. Complex 1 has three functional modules [3]: the N module electron accepting (input) and the Q module ubiquinone reducing (electron output) both located in the hydrophilic arm and the P proton pumping/translocase within the hydrophobic membrane arm. The energy generated by electron transfer within the matrix arm is transduced, by conformational changes in the membrane arm, to pump four protons into the mitochondrial inter-membrane space.

Supercomplex assembly is thought to provide structural and functional advantages to the enzymes of the respiratory chain [4], thereby improving efficiency of the respiratory chain production of ATP, as well as reducing ROS generation [5–7]. More recent work suggests that the respiratory enzymes exist in a solid complex of varying ratios of Complex 1, 3 and 4 to form the supercomplex [8].

Low energy charge and purine quantity in donor liver parenchyma determines poor outcome in transplantation, which is related to mitochondrial function. The application of persufflation, gaseous perfusion with oxygen, to ‘resuscitate’ organs for transplantation has been explored in a number of solid organs [9]. The mitochondrial basis of liver transplant outcome has not been fully elucidated. However, there is some data that shows preservation of Complex 1 is important and improves the quality of cryopreserved human hepatocytes [10]. Additionally, in animal models, Complex 1 activity has been demonstrated to be reduced in both steatosis and increasing CIT [11, 12].

The factors that determine supercomplex assembly and function are still being resolved but it is recognized that mitochondrial metabolic needs, as defined by cell type and physiological status are a determinant [13]. Putative assembly factors for the supercomplexes are being characterized [14]. Of interest and relevant to transplantation, a group of hypoxia induced gene products present in the inner mitochondrial membrane have been found to associate with the supercomplex and contribute to its stability/assembly. These hypoxia assembly factors influence supercomplex levels only, with no effect on individual complex levels and maybe the basis for changing supercomplex organization with oxygen availability [15, 16]. This observation could explain the present finding that no change in subunit composition of Complex 1 was seen on exposure to oxygen.

In conclusion, the presented data on subunit composition of Complex 1 suggest it is fully assembled, as no degradation of subunits associated with the different parts of the enzyme was seen. However, more work is needed to characterize, the factors that define the active/de-active state of Complex 1 and whether Complex 1 assembly, is a critical determinant of donor liver mitochondrial function and transplant outcome.

## Limitations

The number of samples available for analysis and comparison are small. No functional assays have been performed to assess Complex 1 activity. Neither have any co-immunoprecipitation experiments been undertaken to demonstrate protein associations. Ideally, Blue Native polyacrylamide gel electrophoresis (BN-PAGE) to study assembly of Complex 1 combined with in gel measurement of enzyme activity on a larger number of transplanted and non transplanted livers needs to be undertaken.

## Abbreviations

ATP: adenosine triphosphate
A-PSF: anterograde persufflation
DBD: donor after brainstem death
DCD: donor after cardiac death
ETC: electron transport chain
LM: lauryl n-dodecyl β-d-maltoside
LLS: left lateral segment
MTNDI: mitochondrial encoded NADH dehydrogenase I
NDUFB6: NADH dehydrogenase (Complex 1) 1β subcomplex 6
NDUFA10: NADH dehydrogenase (ubiquinone) 1α subcomplex 10
NDUFV2: NADH dehydrogenase (ubiquinone) flavoprotein 2
PBS-T: phosphate buffered saline—0.3% Tween
OXPHOS: oxidative phosphorylation
PVDF: polyvinylidene difluoride
PV: portal vein
ROS: reactive oxygen species
UW: University of Wisconsin Solution
WIT: warm ischemic time
